# Supplementary material for: Comparative genomics provides new insights into the diversity, physiology, and sexuality of the only industrially exploited tremellomycete: Phaffia rhodozyma
Source: BMC Genomics. 2016 Nov 9;17:901. doi: 10.1186/s12864-016-3244-7 (PMC5103461; doi:10.1186/s12864-016-3244-7)
Supplement: Additional file 6: — List of orphan genes with links to PFAM (related to Additional file 1: Table S1). (ZIP 1428 kb) [file 12864_2016_3244_MOESM6_ESM.zip › PFAM_UNIQUE/G05235_P.html]

#                                                                            --- full sequence --- -------------- this domain -------------   hmm coord   ali coord   env coord
  
# target name        accession   tlen query name           accession   qlen   E-value  score  bias   #  of  c-Evalue  i-Evalue  score  bias  from    to  from    to  from    to  acc description of target
  
#------------------- ---------- ----- -------------------- ---------- ----- --------- ------ ----- --- --- --------- --------- ------ ----- ----- ----- ----- ----- ----- ----- ---- ---------------------
  
G05235\_P             -            162 MPLKIP               PF15502.1    151     0.048   13.0  27.1   1   1    0.0015       1.8    7.9  27.1    18   134    22   160     6   161 0.53 - M-phase-specific PLK1-interacting protein  
G05235\_P             -            162 Pro-rich             PF15240.1    179       3.9    6.2  12.7   1   1    0.0037       5.5    5.7  12.7     9   135     7   126     5   148 0.62 - Proline-rich  
G05235\_P             -            162 PT                   PF04886.7     36         1    7.4   4.7   1   2   0.00015      0.45    8.6   2.2     9    24    19    34    17    39 0.83 - PT repeat  
G05235\_P             -            162 PT                   PF04886.7     36         1    7.4   4.7   2   2      0.58   1.7e+03   -2.9   0.0    18    21   106   109   104   109 0.30 - PT repeat
